# Comparison of passive and active motion: effect of myokine irisin on cartilage in knee osteoarthritis rats

**DOI:** 10.3389/fphys.2025.1639174

**Published:** 2025-07-29

**Authors:** Zhengjiao Duan, Hongpan Gao, Xue Xiao, BenXiang He

**Affiliations:** ^1^School of Sports Medicine and Health, Chengdu Sport University, Chengdu, China; ^2^ Affiliated Sport Hospital of Chengdu Sport University, Chengdu, China; ^3^ Sichuan Academy of Chinese Medicine Sciences, Chengdu, China

**Keywords:** knee osteoarthritis, active motion, passive motion, irisin, chondroprotection

## Abstract

**Introduction:**

Knee osteoarthritis (KOA) severely impacts knee joint health. Irisin, a myokine secreted during muscle contraction, varies with different exercise. This study investigates the chondroprotective effects of irisin induced by active and passive motion in a rat model of KOA.

**Methods:**

The rats were randomly allocated into control (CG), model (OAG), passive motion (PAG), and active motion groups (AAG). KOA was induced by intra-articular injections of monosodium iodoacetate (MIA) in all groups except CG. The PAG and AAG group underwent 4 weeks of treadmill exercise or quadriceps femoris muscle electroacupuncture. Changes in irisin, chondrocyte metabolic markers, and inflammatory factors were evaluated using ELISA, histology, immunohistochemistry, and qRT-PCR. Pearson correlation analysis was performed to assess the relationship between irisin and inflammatory factors.

**Results:**

One-week post-modeling, the OAG group showed the lowest hindlimb grip strength and quadriceps weight, chondrocyte number, collagen II expression and irisin levels, while displaying the highest levels of matrix-degrading enzymes (MMP-13, MMP-9, ADAMTS-5) and inflammatory factors (IL-6, TNF-α, IL-1β, IL-18). However, after 4 weeks of intervention, both the PAG and AAG groups reversed these trends. The PAG group exhibited greater improvements in hindlimb grip strength and quadriceps weight compared to the AAG group. HE staining, toluidine blue and safranin O showed smoother cartilage surfaces, increased cartilage thickness and stronger safranin O staining in PAG and AAG groups compared to OAG. Collagen II protein expression was upregulated (P < 0.001), while MMP–13 was downregulated (P < 0.001), and the mRNA levels of MMP-9 and ADAMTS-5 were downregulated (P < 0.001). Moreover, inflammatory factors were lower in PAG than in AAG. Irisin expression was highest in the PAG group, followed by AAG, CG, and OAG group, in serum, synovial fluid and cartilage. Irisin levels were negatively correlated with IL-6, TNF-α, IL-1β, and IL-18.

**Conclusion:**

Both active and passive motion inhibited cartilage degeneration in KOA rats, with passive exercise showing superior effects on muscle strength, irisin secretion, and downregulation of inflammatory cytokines. The chondroprotective and anti-inflammatory effects were positively correlated with increased irisin expression.

## 1 Introduction

Knee Osteoarthritis (KOA) is a degenerative disease of the knee joint. Epidemiological data indicated that approximately 595 million patients worldwide were diagnosed with KOA in 2020, with the prevalence projected to double by 2050 (GBD 2021 Osteoarthritis Collaborators, 2023). This condition is characterized by a series of progressive symptoms, including chronic knee pain, morning stiffness, joint swelling, muscle atrophy and weakness (Cai et al., 2021). KOA is a leading cause of chronic disability among middle-aged and elderly populations (Wang et al., 2022). Given the rising global burden, understanding the pathological mechanisms and developing effective therapeutic strategies for KOA have become urgent priorities.

Currently, the etiology and pathogenesis of KOA remain incompletely understood. Chondrocytes, the sole cell type in articular cartilage, maintain a dynamic balance between matrix synthesis and degradation by regulating the content of the extracellular matrix (ECM) and the activity of chondrocyte-degrading enzymes, such as matrix metalloproteinases (MMPs) (Roseti et al., 2019). An imbalance between cartilage regeneration and degradation can lead to the depletion of matrix components and irreversible cartilage damage (Hall, 2019). Inflammatory cytokines play a pivotal role in disrupting this balance. For instance, interleukin-1β (IL-1β) and tumor necrosis factor-α (TNF-α) activate the NF-κB signaling pathway, promoting the expression of MMP-13 and ADAMTS-5, which degrade type II collagen and proteoglycans (Zhang et al., 2024). Interleukin-6 (IL-6) exacerbates inflammation through the JAK-STAT pathway, while interleukin-18 (IL-18) synergizes with IL-1β to induce chondrocyte apoptosis and senescence (Liao et al., 2022). These cytokines form a pro-inflammatory cascade: cartilage matrix degradation releases micro-osteophytes and particles, which activate macrophages in the synovial fluid to secrete more inflammatory mediators, further stimulating chondrocytes to produce degrading enzymes (Ji et al., 2019; Mora et al., 2018).

Given the complex pathogenesis of KOA, exploring interventions that can modulate pathological processes becomes crucial. Exercise therapy is an important part of the non-pharmacological treatment of KOA (Mo et al., 2023). For patients with KOA who are not surgical candidates, scientifically designed and appropriate exercise regimens can reduce systemic levels of inflammatory mediators (Conroy, 2024), mitigate chondrocyte senescence (Blank et al., 2024), decrease chondrocyte apoptosis (Schmidt et al., 2017), alleviate pain, improve joint function, enhance joint stability, and increase muscle strength (Callegari et al., 2023).

The exercise modes for KOA treatment can be categorized into active and passive exercises. Active exercises include daily walking on flat ground, aquatic exercise and muscle strength training (e.g., quadriceps femoris) (Greene et al., 2025). Moderate-intensity exercise therapy has been shown to have a positive impact on cartilage matrix repair compared to low- or high-intensity exercise (Bricca et al., 2017). Passive body movement is more suitable for individuals unable to perform active exercise, such as elderly patients with osteoporosis who have weak muscle strength and are at risk of falls and fractures. For these patients, interventions such as isokinetic muscle strength training devices and electro-acupuncture stimulation to induce muscle contraction are appropriate. Continuous passive motion improved function after angular-stable plate osteosynthesis of proximal humerus fractures (Tille et al., 2024).

One of the potential mechanisms of exercise therapy in KOA is myokines. Irisin, a 112-amino acid peptide derived from fibronectin type III domain-containing protein 5 (FNDC5), is primarily secreted during muscle contraction and exhibits high conservation across mammalian species (Boström et al., 2012; Lai et al., 2024). It has demonstrated beneficial effects in several chronic diseases (Martinez Munoz et al., 2018). Recently, studies finds that the emerging myokine irisin is linked to bone and cartilage health problems. Serum irisin levels are lower in individuals with sarcopenia compared to healthy individuals (Coelho-Junior et al., 2019). Lower irisin expression has been detected in chondrocytes of KOA patients, and low irisin levels are positively correlated with the high OARSI score and subchondral bone loss (Jang et al., 2021). *In vitro* studies have shown that irisin treatment of IL-1β-induced inflammatory chondrocytes significantly reduces the expression of pro-inflammatory mediators and inhibits chondrocyte degradation (Li et al., 2021). Irisin regulates OA by inhibiting MAPK signaling pathway, NF-kB signaling pathway and inflammation-mediated oxidative stress (Zhao et al., 2023). It improves chondrocyte metabolism, enhances chondrocyte activity, and protects articular cartilage from inflammatory responses, thereby alleviating OA progression.

Although exercise can induce the expression of irisin, which exhibits anti-inflammation role in KOA, different types of exercise have varying effects on irisin induction (Jandova et al., 2021), and muscle mass and muscle health status can also affect the secretion of irisin (Shang et al., 2024). Besides, current research lacks a comprehensive comparison of the passive and active exercise modes effect on KOA. Therefore, in this study, moderate-intensity treadmill exercise was employed as the active exercise mode, while electropuncture of the quadriceps femoris to induce muscle contraction was used as the passive exercise mode. The effects of the two exercise modes, active exercise and passive exercise, on cartilage repair, irisin, and inflammatory factors in KOA rats were investigated, with the aim of providing a research basis and theoretical foundation for exercise therapy in KOA.

## 2 Methods and materials

### 2.1 Experimental animals

This study was approved by the Animal Experiment Ethics Committee of Chengdu Sport University (approval No. 202213). All animal experiments were carried out in accordance with the U.K. Animals (Scientific Procedures) Act, 1986 and associated guidelines. Thirty-two Sprague-Dawley (SD) rats (8 weeks old, female, 180–220 g) were purchased from SiBeiFu Bioscience Co. Ltd. (Beijing, China) (certificate no. SYXK-2023-0100). Animal housing environment: The light-dark cycle was set at 12 h of light and 12 h of darkness. The temperature was maintained at 22°C ± 2°C, and the relative humidity was kept within the range of 40%–60%. No more than four rats were housed in each cage.

### 2.2 OA model and exercise treatment protocols

Model group (n = 24) was anesthetized by intraperitoneal injection of chloral hydrate (8% concentration, 2 mL/100 g). Then, injecting sodium iodoacetate (MIA, 0.5 mg per cavity in 50 μL sterile saline) (Tantowi et al., 2020) into the knee joint cavity of both hind limbs. The control group (CG, n = 8) received an equal volume of saline. After 1 week of modelling, the model group were randomly divided into 3 groups: OA model group (OAG, n = 8), passive activity group (PAG, n = 8), active activity group (PAG, n = 8).

The PAG received electropuncture to induce quadriceps femoris contraction (Cheng et al., 2024). The rats were fixed, and the electrical stimulation parameters were set as follows: 1–3 mA, 1 Hz, 15 min per session. The treatment was administered 4 times a day, for 5 days a week. The AAG underwent moderate-intensity treadmill training on the animal treadmill exercise platform (ZH-PT, Zhongshi Dichuang Corp., Beijing, China). The treadmill was set at 19.3 m per minute, 5°inclination, with each training session lasting 60 min. The training was conducted once a day, 5 days a week (Hu et al., 2024). The CG and OAG were fed with normal feed and received no treatment. All interventions were carried out for 4 weeks.

### 2.3 Hind limb grip strength

Hind limb grip strength was measured by rat grip strength meter (YR22-YLS-13A, Yaokun Corp., Anhui, China) on the 7th day after establishment of the KOA model (baseline) and after the 4 weeks treatment.

### 2.4 Tissue samples

After the final intervention, an overdose of chloral hydrate (10% concentration, 2 mL/100 g) was injected into the rats’ peritoneum for anesthesia. Tissues were collected from Sprague-Dawley (SD) rats in each group. Blood was drawn from the aorta abdominalis, 2–2.5 mL blood for each rat. Then blood was centrifuged at 4°C, 3,000 rpm for 15 min to obtain serum. We perfused 0.1 mL phosphate-buffered saline (PBS) into the knee joint and suctioned synovial fluid (SF). The rats were dissected to collect quadriceps femoris, knee joints and cartilage.

### 2.5 Pathological examination

Knee joints were fixed in 4% paraformaldehyde solution, decalcified in 20% EDTA solution and embedded in paraffin wax. The specimens were then sectioned in the sagittal position and stained with hematoxylin and eosin (HE) toluidine blue, and safranin O staining. The degree of cartilage was assessed by Mankin’s score (Supplementary File 1).

### 2.6 Enzyme-linked immunosorbent assay (ELISA)

The level of irisin, IL-6 and TNF-α in the serum and SF were examined by enzyme-linked immunosorbent assay (ELISA) kit (ZC-37565, ZC-36404, ZC-37624 Zci Bio, Shanghai, China) on a spectrophotometric reader at 450 nm. All operations were complied with instructions. The sensitivities were 0.1 ng/mL for irisin, 1.0 pg/mL for IL-6, and 1.0 pg/mL for TNF-α, respectively, with intra-assay and inter-assay coefficients of variation (CVs) < 10%.

### 2.7 Immunohistochemistry

The immunohistochemistry steps were as follows: The sections were deparaffinized and hydrated; antigen retrieval was performed using an enzymatic method; an endogenous peroxidase blocker was used to inhibit the activity of endogenous peroxidase; the sections were blocked with bovine serum albumin (BSA). Sections were then incubated with the following primary antibodies: anti-FNDC5 (ab174833, 1:100, Abcam, Cambridge, MA, United States), anti-collagen II (ab34712, 1:100, Abcam), and anti-MM-13 (18165-1-AP, 1:50, Proteintech Group, Rosemount, IL, United States). After washing away the primary antibodies, sections were treated with IHC Detection Reagent (HRP, rabbit; 8,114, Cell Signaling Technology). A DAB chromogenic reaction was performed, followed by counterstaining of cell nuclei. Sections were then dehydrated and mounted with coverslips. Images of the sections were captured using a digital microscope (BA400, Motic China Group Co., Ltd., Fujian, China). The percentage of positive cells was analyzed using Image-Pro Plus version 6.0 software. We quantified the positive cells based on area. Positive cells were defined as those with brown-yellow cytoplasmic/nuclear staining (OD intensity > background, calibrated by negative controls), with 5 × 200 fields analyzed to calculate positive area percentage relative to total tissue.

### 2.8 Quantitative reverse transcription polymerase chain reaction (qRT-PCR)

Total RNA was extracted from articular cartilage using the RNA Extraction Kit (Biosharp Biotech Co., Ltd., Beijing, China). Then, 1 μg RNA was reversed transcribed into cDNA using Takara RNA PCR Kit (AMV) Ver. 3.0 (Takara Bio, Ohtsu, Japan). The primers utilized for the quantification of matrix metalloproteinases-9 (MMP-9), a disintegrin and metalloprotease with thrombospondin type 5 motif (ADAMTS-5), interleukin-1β(IL-1β) and interleukin-18 (IL-18) were designed by and purchased from Sangon, China (Table 1). The RT-PCR reactions were performed using the SYBR Green PCR kit (Vazyme Biotech Co., Ltd., Nanjing, China) with the Applied Biosystems 7500 Real-Time PCR System. Each sample was run in triplicate. Relative mRNA expression was calculated using the 2^−ΔΔCT^ method with β-actin as reference.

**TABLE 1:** The primers sequences for qRT-PCR.

Primer name	Upstream	Downstream
IL-1β	aatctcacagcagcatctcgacaag	tccacgggcaagacataggtagc
IL-18	cgaccgaacagccaacgaatcc	gtcacagccagtcctcttacttcac
MMP-9	tcctcttggtggctgactcttc	ttctcgatgcttgcatgactgtac
ADAMTS-5	tcctcttggtggctgactcttcc	tggttctcgatgcttgcatgactg
β-actin	gggaaatcgtgcgtgacatt	gcggcagtggccatctc

### 2.9 Statistical analysis

All data were expressed as means ± standard error of the mean (SEM) using GraphPad Prism 10.0.0. If the data satisfy the normal distribution and homogeneity of variance, one-way analysis of variance (One-way ANOVA) was used to test the significance of the differences. Otherwise, the Kruskal-Wallis test was applied. The Tukey statistical method is employed for *post hoc* multiple comparisons. For the correlation analysis, the Pearson correlation test was adopted. The significance level is set at 0.05.

## 3 Results

### 3.1 Both motions repaired degenerative damage of the articular surface in KOA rats

**FIGURE 1:**
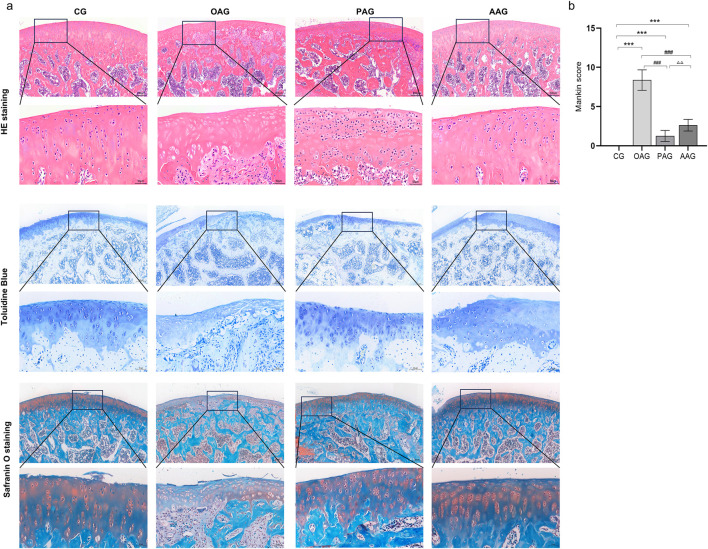
He staining, toluidine blue and safranin O staining of articular cartilage (Magnification:200μm and 50 μm). CG: control group; OAG: KOA model group; PAG: passive motion group; AAG: active motion group. Compared with CG, *P < 0.05, **P < 0.01, ***P < 0.001; Compared with OAG, ^#^ *P* < 0.05, ^##^ *P* < 0.01, ^###^ *P* < 0.001; Compared with PAG, ^△^ *P* < 0.05, ^△△^ *P* < 0.01, ^△△△^ *P* < 0.001; ns: not significant.

The H&E staining, toluidine blue and safranin O staining results of the knee joint revealed that the cartilage structures on the surfaces of femur and tibia in CG exhibited a healthy and physiological state. The joint surfaces were smooth, the cell morphology was typical, and the tidal lines were clearly demarcated, collectively indicating normal cartilage integrity and organization. Safranin O staining showed a large number of proteoglycans were heterochromatically stained bright red and the subchondral bone cortex and bone were stained green. OAG distinct manifestations of cartilage damage were observed, including cracks on the cartilage surface, disarrayed chondrocyte arrangement, disrupted tidal lines, a thinned cartilage layer and weak safranin O staining. These pathological features collectively validated the successful establishment of the KOA model. Conversely, the PAG and the AAG showed reduced cartilage injury. In PAG, the cartilage surface was slightly rough, and the chondrocytes were mildly deformed. The matrix staining was relatively uniform. The thickness of the cartilage layer and the degree of matrix degeneration were reduced compared with those in the OAG group. Safranin O staining was stronger than OAG group. The degree of injury in the AAG was improved compared with that in the OAG. The cartilage surface was relatively smooth, the cartilage layer was thickened, the number of chondrocytes increased, and the tidal line was relatively clear and complete. Safranin O staining intensity was greater compared to the OAG group but less than CG group (Figure 1). Compared with the OAG, the Mankin scores increased in the PAG and AAG (*P < 0.001*) (Figure 1b).

### 3.2 Passive motion strengthened hindlimb grip and quadriceps femoris of the KOA rat model

**FIGURE 2:**
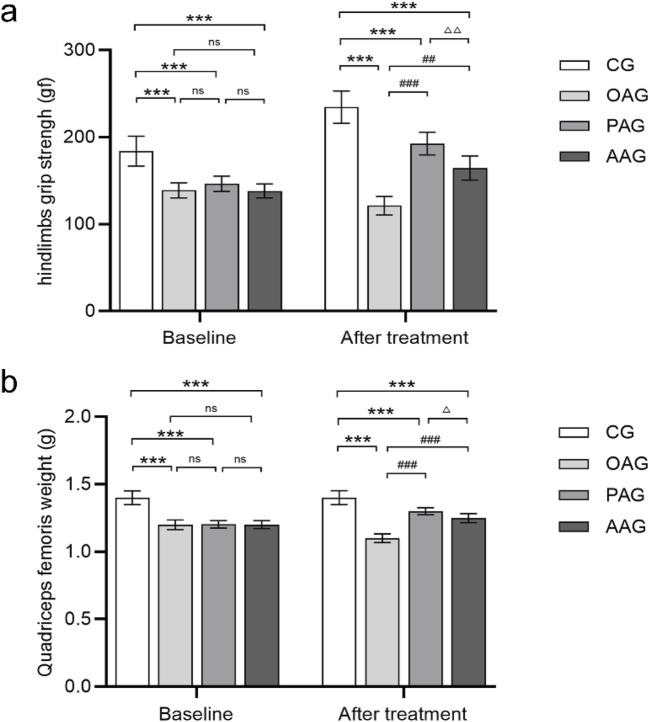
Passive motion increased grip strength of the hindlimbs and quadriceps femoris weight. **(a)** The grip strength of the rats was measured for all groups at two-time nodes. **(b)** Quadriceps femoris weight was measured for all groups at two-time nodes. Baseline was the 7th day after establishment of the KOA model. Time point of “after treatment” was after 4 weeks exercise intervention and before dissection. CG: control group; OAG: KOA model group; PAG: passive motion group; AAG: active motion group. Compared with CG, *P < 0.05, **P < 0.01, ***P < 0.001; Compared with OAG, ^#^ *P* < 0.05, ^##^ *P* < 0.01, ^###^ *P* < 0.001; Compared with PAG, ^△^ *P* < 0.05, ^△△^ *P* < 0.01, ^△△△^ *P* < 0.001; ns: not significant.

The comparisons of quadriceps femoris weight and hindlimb grip strength among groups are presented in Figures 2a,b. In the OAG, severe atrophy of the quadriceps femoris was evident, with the lowest muscle weight and the weakest hindlimb grip strength. Both exercise intervention methods delayed the reduction in quadriceps femoris weight and the decline in hindlimb grip strength, showing statistically significant differences compared with the OAG (*P < 0.001*). Compared with the AAG, the PAG had a more positive impact on quadriceps femoris weight and hindlimb grip strength (*P < 0.001*).

### 3.3 Passive activity improved irisin expression of the KOA rat model

**FIGURE 3:**
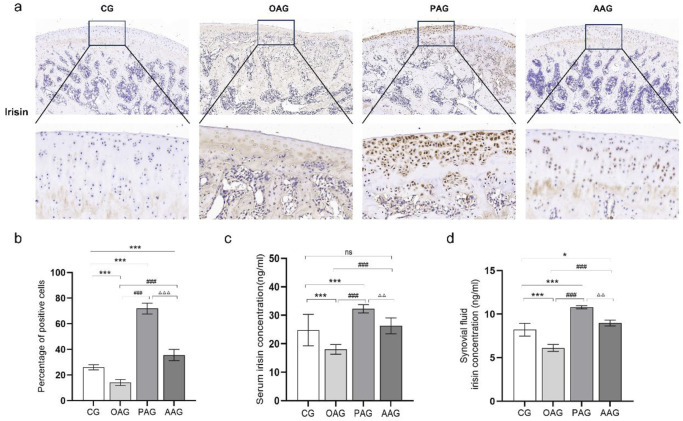
Passive motion significantly induced irisin expression (Magnification:100 μm and 50 μm). **(a,b)** Immunohistochemistry results of irisin in cartilage across all groups. **(c)** Irisin expression in serum. **(d)** Irisin expression in synovial fluid. CG: control group; OAG: KOA model group; PAG: passive motion group; AAG: active motion group. Compared with CG, *P < 0.05, **P < 0.01, ***P < 0.001; Compared with OAG, ^#^ *P* < 0.05, ^##^ *P* < 0.01, ^###^ *P* < 0.001; Compared with PAG, ^△^ *P* < 0.05, ^△△^ *P* < 0.01, ^△△△^ *P* < 0.001; ns: not significant.

ELISA results showed that compared with the CG, the contents of irisin in the serum and synovial fluid in the OAG were significantly decreased (*P < 0.001*). When compared with the OAG, the protein expression of irisin in the serum and synovial fluid increased in both the PAG and the AAG (*P < 0.001*). Moreover, the PAG was more effective than the AAG in inducing the production of irisin protein in the serum and synovial fluid (Figures 3c,d). The immunohistochemical results supported the similar trend. The OAG had the fewest positive cells labeled with irisin. In comparison with the OAG, both the PAG and the AAG showed a significant increase in the tan staining of irisin (*P < 0.001*). The proportion of positive cells in the PAG was larger than that in the AAG (Figures 3a,b).

### 3.4 Both exercise intervention alleviated cartilage damage in KOA rats

**FIGURE 4:**
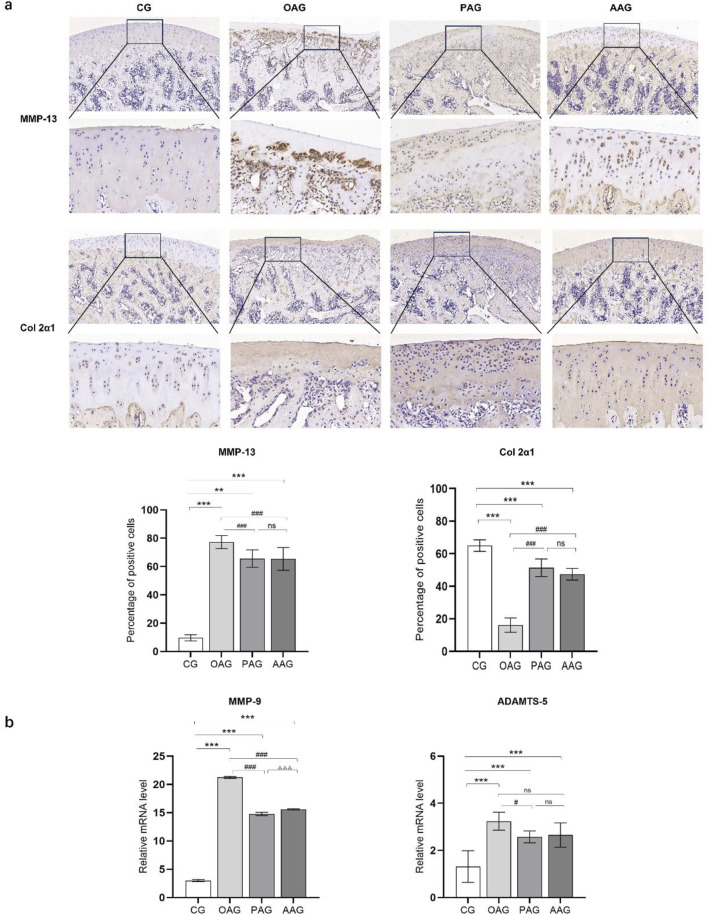
Both exercise intervention protected the cartilage in KOA rats. **(a)** Immunohistochemistry results of MMP-13 and Col 2a1 (Magnification:100 μm and 20 μm). **(b)** The mRNA level of MMP-9 and ADAMTS-5. CG: control group; OAG: KOA model group; PAG: passive motion group; AAG: active motion group. Compared with CG, *P < 0.05, **P < 0.01, ***P < 0.001; Compared with OAG, ^#^ *P* < 0.05, ^##^ *P* < 0.01, ^###^ *P* < 0.001; Compared with PAG, ^△^ *P* < 0.05, ^△△^ *P* < 0.01, ^△△△^ *P* < 0.001; ns: not significant.

The cartilage repair situation of KOA rats was observed through immunohistochemical staining of MMP-13 and type II collagen. The OAG had the fewest positive cells labeled with type II collagen and the most positive cells labeled with MMP-13. Compared with the OAG, both the PAG and the AAG showed a significant increase in the tan staining of type II collagen and a remarkable reduction in the proportion of MMP-13 positive cells (*P < 0.001*). There was no significant difference in the expression of MMP-13 and type II collagen between the PAG and the AAG (Figure 4a). The qRT-PCR results showed that the mRNA levels of ADAMTS-5 and MMP-9 in the OAG were significantly upregulated (*P < 0.001*). When compared with the OAG, the mRNA levels of MMP-13 and MMP-9 in the PAG and the AAG were reversed. Although there was no significant difference between the PAG and the AAG, the PAG showed a trend of better repair effect than the AAG (Figure 4b).

### 3.5 Passive exercise intervention decrease inflammatory factors expression in KOA rats

**FIGURE 5:**
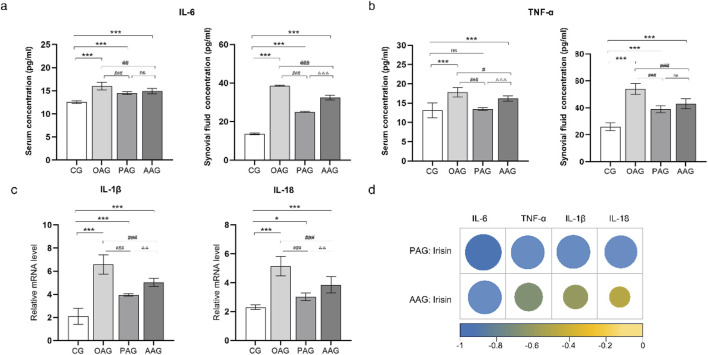
Passive motion significantly reduced inflammatory factors expression. **(a)** IL-6 level in serum and synovial fluid. **(b)** TNF-α level in serum and synovial fluid. **(c)** The mRNA level of IL-1β and IL-18 in cartilage. **(d)** The circle represented pearson product-moment correlation coefficient. The levels of irisin in the synovial fluid were negatively correlated with the expressions of TNF-α, IL-6. The levels of irisin in the cartilage were negatively correlated with the expressions of IL-1β and IL-18. CG: control group; OAG: KOA model group; PAG: passive motion group; AAG: active motion group. Compared with CG, *P < 0.05, **P < 0.01, ***P < 0.001; Compared with OAG, ^#^ *P* < 0.05, ^##^ *P* < 0.01, ^###^ *P* < 0.001; Compared with PAG, ^△^ *P* < 0.05, ^△△^ *P* < 0.01, ^△△△^ *P* < 0.001; ns: not significant.

The levels of TNF-α and IL-6 in the serum and synovial fluid of KOA rats were measured using ELISA. The results indicated that the lowest levels of TNF-α and IL-6 were found in the serum and synovial fluid of the CG. In comparison to the CG, the levels of TNF-α and IL-6 in the serum of other groups were elevated, although no significant differences were observed among these groups. In the synovial fluid, the levels of TNF-α and IL-6 were significantly upregulated in the OAG compared to the CG (*P < 0.001*). The PAG and AAG groups demonstrated anti-inflammatory effects in the synovial fluid, with significantly downregulated levels of TNF-α and IL-6 compared to the OAG (*P < 0.001*). Notably, the levels of these inflammatory factors were lower in the PAG group than in the AAG group (Figures 5a,b). At the gene level, the mRNA expressions of IL-1β and IL-18 were significantly upregulated in the CG (*P < 0.001*). The PAG and AAG effectively reversed this upregulation and inhibited the mRNA expressions of IL-1β and IL-18, with the PAG showing a more pronounced inhibitory effect than the AAG (*P < 0.001*) (Figures 5c,d). Additionally, the levels of inflammatory factors in the local knee joint, specifically in the synovial fluid and cartilage, were found to be higher than those in the systemic circulation.

### 3.6 Negative correlation between irisin and inflammatory factors

In KOA rats undergoing the two types of exercise, the expression levels of irisin in the synovial fluid were negatively correlated with the expressions of inflammatory factors TNF-α, IL-6. The levels of irisin in the cartilage were negatively correlated with the IL-1β and IL-18 (Figure 5e; Supplementary Figure S1).

## 4 Discussion

In this study, we focused on irisin to investigate the mechanisms underlying the effects of two different exercise modalities on KOA. Employing a comprehensive suite of detection methods, including HE staining, toluidine blue staining, safranin O staining, immunohistochemistry, ELISA, and qRT-PCR, we uncovered that passive motion, specifically electrical stimulation-induced contraction of the quadriceps femoris muscle, was more effective in improving muscle quality and inducing irisin secretion. This exercise mode also significantly downregulated the expression of inflammatory factors in the KOA rats. Both active and passive exercises demonstrated comparable efficacy in repairing cartilage damage, as evidenced by the upregulation of type II collagen and the suppression of matrix-degrading enzymes such as MMP-13, MMP-9, and ADAMTS-5. The therapeutic effects of exercise intervention on KOA may be attributed to the upregulation of irisin, which subsequently inhibits the expression of inflammatory factors, thereby mitigating the inflammatory cascade and promoting cartilage repair.

Exercise intervention serves as a crucial component in the management of KOA. The regulation of KOA by myokines released from muscles during exercise has consistently been a key focus of research. Irisin is abundant in skeletal muscle tissue. During exercise, mechanical stress triggers cytoskeletal remodeling, activating the integrin-FAK-PI3K/Akt-PGC-1α pathway to upregulate FNDC5 transcription and irisin secretion (Moreno-Navarrete et al., 2013). Numerous studies have demonstrated that exercise effectively induces irisin expression (Guo et al., 2024), with resistance training showing a more pronounced effect compared to moderate-intensity aerobic exercise (Tsuchiya et al., 2015). However, research on the induction of irisin by passive exercise remains limited.

Our study demonstrated that passive exercise mode significantly enhancing irisin production. Electropuncture of the quadriceps femoris muscle exerted positive effects on muscle quality and hindlimb grip strength in KOA rats. This might be attributed to the fact that pain, a common symptom of KOA, often led to reduced exercise motivation, decreased irisin secretion, and subsequent muscle atrophy (Luan et al., 2022). The significant decline in quadriceps femoris weight and hindlimb grip strength observed in the OAG group corroborated this finding. Electrical stimulation not only alleviated pain but also sustained muscle contraction, thereby effectively improving muscle weight and grip strength (Rahmati et al., 2021). In contrast, treadmill exercise, which involved the engagement of multiple muscle groups (Hirjaková et al., 2020), resulted in compensatory muscle activation, leading to relatively lower irisin secretion from the quadriceps femoris muscle. Exercise can also activate mechanosensitive ion channels, such as Piezo1 and TRPV4 on the muscle cell membrane, causing a transient increase in intracellular calcium ion concentration (Nunes et al., 2022; Watanabe et al., 2003; Liu et al., 2017). Calcium ions, as essential second messengers, activate calmodulin (CaM), which in turn initiates the downstream calcineurin (CaN)-nuclear factor of activated T cells (NFAT) signaling pathway, promoting the expression of genes related to muscle growth and repair, such as MyoD and Myogenin (Ehlers et al., 2014; Witwicka et al., 2019).

In terms of cartilage repair, both active and passive motion demonstrated positive effects. Histopathological, immunohistochemical, and qRT-PCR analyses revealed that following exercise intervention, the number of chondrocytes increased, and cartilage surface defects were alleviated and proteoglycans were increased compared to the OAG group. The expression of type II collagen was upregulated, while the expression of MMP-13, MMP-9 and ADAMTS-5 was significantly downregulated. The upregulation of type II collagen indicated enhanced anabolic metabolism of the cartilage matrix, facilitating cartilage repair and reconstruction (J et al., 2024). Conversely, the downregulation of MMPs effectively inhibited catabolic metabolism, maintained the balance between cartilage matrix synthesis and degradation, thereby promoted cartilage repair (Deng et al., 2018). Our findings were consistent with previous studies. Liu et al. (2023) observed that moderate-intensity treadmill exercise significantly reduced joint surface sclerosis and osteophyte formation in KOA rats. Research has found that mechanical stimulation improved chondrocyte function (Shadi et al., 2022). Notably, our study found no significant differences in cartilage repair efficacy between the two exercise modes. This result provided greater flexibility and feasibility for clinicians to select appropriate exercise therapies based on individual patient conditions. For patients with adequate mobility, active exercise can be the preferred option, while for those with limited mobility or weak muscle strength, externally induced muscle contraction can also exert therapeutic effects.

Although cartilage pathology between the two exercise modes showed similar outcome, the underlying molecular mechanism gave another side perspective. Both exercise modes in our study effectively downregulated the expression of inflammatory factors. Elevated levels of inflammatory factors were detected in the abdominal artery blood of KOA rats, confirming that KOA induces systemic chronic inflammation, consistent with previous studies (Hamedi-Shahraki et al., 2025). Interleukin family members, such as IL-1β, IL-6, and IL-18, are pro-inflammatory cytokines that amplify inflammatory responses and contribute to joint destruction and remodeling through the stimulation of matrix-degrading enzymes (Yuce et al., 2021). TNF-α, primarily synthesized and secreted by chondrocytes, macrophages, and fibroblasts, upregulates the expression of MMPs, accelerating the catabolic metabolism of cartilage matrix components such as type II collagen and proteoglycans (Park et al., 2021). TNF-α also activates osteoclast precursor cells, promoting osteoclast differentiation and bone resorption, thereby exacerbating abnormal subchondral bone remodeling (Yao et al., 2021).

Previous studies have demonstrated the anti-inflammatory properties of irisin. In obese mouse models, irisin administration alleviated inflammation in adipose tissue and improved insulin sensitivity (Slate-Romano et al., 2022). Irisin pretreatment reduced inflammation cell infiltration and inflammatory factor expression in the lungs of LPS-induced mice (Han et al., 2023). Irisin exerts its anti-inflammatory effects by inhibiting the activation of NF-κB, reducing its translocation to the nucleus, and downregulating the transcription and expression of inflammatory factors such as TNF-α and IL-6 (Vadalà et al., 2020). Inflammation is often accompanied by increased oxidative stress, which irisin can mitigate by activating intracellular antioxidant pathways. Irisin upregulates the expression and activity of antioxidant enzymes such as superoxide dismutase (SOD) and glutathione peroxidase (GPx), reducing the generation of reactive oxygen species (ROS) and alleviating oxidative stress damage, thereby indirectly inhibiting inflammatory responses (Mazur-Bialy and Pocheć, 2021). In our study, a negative correlation was observed between irisin expression and inflammatory factors including TNF-α, IL-6, IL-1β, and IL-18, further supporting the endogenous protective role of irisin in the pathogenesis of KOA. It appeared that irisin might act as a key regulatory factor, alleviating KOA symptoms in rats by inhibiting inflammatory factor expression. Interestingly, although the anti-inflammatory effect was more pronounced in the PAG group compared to the AAG group, no significant differences were observed in cartilage repair efficacy between the two groups. This discrepancy may be attributed to the complex pathogenesis of KOA, which involves the interplay of various cytokines, growth factors, and extracellular matrix components (Aihaiti et al., 2025). Compared to passive muscle contraction, treadmill exercise also induces knee flexion and extension, facilitating the exchange of substances within the knee joint cavity through diffusion (Takahashi et al., 2023). In addition to myokines, the mechanisms of exercise therapy also include biomechanics and local microenvironment. Previous study confirmed that immobilization can induce cartilage degeneration, whereas exercise can enhance the repair capacity of degenerated articular cartilage, promotes the morphological recovery of joint cartilage, redistributes stress across the weight-bearing joint surface, and restores joint stability (Dieterle et al., 2021). Active exercise applies dynamic compressive forces that promote chondrocyte mechanotransduction, upregulate type II collagen, and downregulate MMP13 (Nomura et al., 2024). Conversely, passive stimulation reduces shear stress on cartilage, offering indirect protection (Wei and Dai, 2021). These distinct biomechanical mechanisms collectively contribute to joint stability and cartilage recovery. Furthermore, exercise can alter the chemical environment of cartilage. Modulating the immune activity of synovial tissue by reducing the secretion of pro-inflammatory factors from synovial fibroblasts or by promoting their release of anti-inflammatory mediators, thereby ameliorating the local inflammatory microenvironment in the knee joint (Jiang et al., 2023). Therefore, future research should focus on elucidating the specific molecular mechanisms by which exercise regulates the knee joint microenvironment to provide a more robust theoretical basis for the development of precision exercise rehabilitation protocols.

Unlike our study, which observed the expression of irisin through two exercise modes, researchers injected irisin into the articular cavity of KOA rats and found that it alleviated oxidative stress, cartilage erosion, and synovitis by regulating mitochondrial integrity and autophagy (Wang et al., 2020). Jia et al. preincubated chondrocytes with recombinant irisin before treatment with IL-1β, while the dosage was based on the synovial fluid irisin concentrations in rats. They found irisin protected chondrocyte pyroptosis through P13K/Akt/NF-κB signal pathway (Liu et al., 2023). Regarding the clinical application of irisin, it is currently mostly employed as a biomarker. Researchers have investigated different exercise interventions in type 2 diabetic patients, measuring levels of fibroblast growth factor 21 (FGF21), irisin, myostatin (MSTN), and follistatin (FST) in blood samples, and identified the concurrent training that increased irisin and FST while decreasing MSTN as the optimal approach (Motahari Rad et al., 2023). Additional studies have shown that serum irisin expression raised following exercise intervention in depressed patients, correlating with significant alleviation of depressive symptoms (Bilek et al., 2022). After vitamin D supplementation, researchers observed elevated serum irisin levels alongside increased thyroid-stimulating hormone in women with subclinical hypothyroidism, suggesting a link between irisin secretion and thyroid function (Safari et al., 2023). However, clinical therapeutic applications of irisin remain unexplored. Challenges include its complex, incompletely elucidated mechanism of action, as well as its low bioavailability and short half-life, which may necessitate frequent injections or oral administration to maintain effective concentrations—factors that could markedly reduce patient compliance and potentially increase the risk of adverse effects (Paoletti and Coccurello, 2024).

In this study, we adopted treadmill exercise as the active exercise mode and electropuncture of the quadriceps femoris muscle as the passive exercise mode. We found that both exercises effectively improved cartilage damage in KOA rats and downregulated the expression of inflammation in synovial fluid and cartilage. Notably, the direct induction of quadriceps femoris contraction was more effective in inducing irisin production and improving quadriceps femoris quality. A negative correlation was observed between irisin and inflammatory factors, indicating the anti-inflammatory effects of irisin. There were, however, some limitations in this study. First, the selection of exercise modes in this study, namely animal treadmill exercise and electrical stimulation-induced contraction of the quadriceps femoris muscle, might limit the generalizability of the findings. In clinical practice, a wide variety of exercise modes are employed, and their differential impacts on KOA remain to be explored. Second, the experimental intervention lasted only 4 weeks, precluding the assessment of long-term effects. Given that KOA is a chronic disease requiring prolonged intervention, the long-term effects of exercise therapy on cartilage protection, irisin secretion regulation, and inflammation suppression remain uncertain. Additionally, potential issues arising from long-term exercise intervention could not be evaluated. Finally, although a negative correlation between irisin and inflammatory factors was observed, the specific mechanisms underlying the regulation of inflammatory factor expression and the influence on chondrocyte metabolism by irisin were not thoroughly investigated. Future studies should be conducted to explore the precise signaling pathways through which irisin modulates inflammatory factor expression and affects chondrocyte metabolism.

## 5 Conclusion

Both treadmill exercise and electropuncture of the quadriceps femoris muscle effectively promoted cartilage repair and protected the knee joints, as evidenced by the increased number of chondrocytes, upregulation of type II collagen, inhibition of MMP-13, MMP-9, and ADAMTS-5 expression, and downregulation of IL-6, IL-1β, IL-18, and TNF-α. The passive exercise mode, which directly induced contraction of the quadriceps femoris through electrical stimulation, was more effective in inducing irisin secretion, improving muscle quality compromised by KOA, and exerting anti-inflammatory effects. The exercise-induced irisin may alleviate KOA through its anti-inflammatory effects.

## Data Availability

The original contributions presented in the study are included in the article/Supplementary Material, further inquiries can be directed to the corresponding authors.

## Supplementary material

SUPPLEMENTARY FIGURE S1 Scatter plot showed the negative correlation between irisin and IL-6, TNF-α, IL-β and IL-18.
