# Supplementary material for: Comparison of passive and active motion: effect of myokine irisin on cartilage in knee osteoarthritis rats
Source: Front Physiol. 2025 Jul 29;16:1639174. doi: 10.3389/fphys.2025.1639174 (PMC12339569; doi:10.3389/fphys.2025.1639174)
Supplement: Supplementary file 1 [file Table1.docx]

**Mankin’s Score**

1. **Cartilage Structure Scoring**
   1. **0 point**: Normal cartilage structure. The cartilage surface is smooth, and there are no visible abnormalities in the layers of cartilage, such as the superficial, transitional, radial, and calcified layers. The overall architecture of the cartilage is intact, and the chondrocytes are evenly distributed within their lacunae.
   2. **1 point**: Surface irregularities are present. The cartilage surface may appear slightly rough, but there are no deep fissures or significant disruptions. The integrity of the cartilage layers is still relatively maintained, and chondrocyte distribution is mostly normal.
   3. **2 points**: Pannus formation and surface irregularities. In addition to the rough surface, there is the presence of pannus, which is a layer of abnormal tissue that can grow over the cartilage. This pannus can contribute to further cartilage damage and degradation.
   4. **3 points**: Fissures extend into the transitional layer. The cartilage has developed cracks that penetrate into the transitional layer, which is located between the superficial and radial layers. These fissures can disrupt the normal structure and function of the cartilage, potentially leading to further breakdown.
   5. **4 points**: Fissures extend into the radial layer. The fissures are deeper and now reach the radial layer of the cartilage. This layer is important for maintaining the mechanical strength of the cartilage, and the presence of fissures here can significantly compromise the cartilage's ability to withstand stress.
   6. **5 points**: Fissures extend into the calcified layer. The damage is even more severe, with fissures reaching the calcified layer of the cartilage. This layer provides additional support to the cartilage, and its disruption can lead to instability and accelerated cartilage degeneration.
   7. **6 points**: Complete structural destruction. The cartilage has lost its normal architecture, and there is significant damage to all layers. Chondrocytes may be absent or severely damaged, and the overall integrity of the cartilage is severely compromised.
2. **Chondrocyte Scoring**
   1. **0 point**: Normal chondrocytes. The chondrocytes are evenly distributed within the cartilage matrix, and their morphology is normal. They are actively producing the components of the extracellular matrix, such as collagen and proteoglycans, to maintain the health of the cartilage.
   2. **1 point**: Diffuse cell increases. There is a slight increase in the number of chondrocytes throughout the cartilage. This may be a sign of the cartilage's attempt to repair itself in response to early damage or stress.
   3. **2 points**: Local cell increase. Instead of a diffuse increase, there are areas where the chondrocytes are clustered together, forming local regions of higher cell density. This clustering can be an indication of more significant damage or an abnormal response to injury.
   4. **3 points**: Marked reduction in cell number. The number of chondrocytes is significantly decreased. This can be due to cell death, which may be caused by factors such as inflammation, oxidative stress, or mechanical trauma. A decrease in chondrocytes impairs the cartilage's ability to maintain and repair itself.
3. **Cartilage Matrix Staining Scoring**
   1. **0 point**: Normal staining. When stained with appropriate dyes, the cartilage matrix shows normal coloration. This indicates that the proteoglycan content in the matrix is normal, and the matrix has its proper composition and structure.
   2. **1 point**: Slight reduction in staining. The staining intensity is slightly less than normal, suggesting a minor decrease in the proteoglycan content of the cartilage matrix. Proteoglycans are important for maintaining the cartilage's water-holding capacity and its ability to resist compression.
   3. **2 points**: Moderate reduction in staining. There is a more noticeable decrease in staining, indicating a moderate loss of proteoglycans. This can lead to a decrease in the cartilage's elasticity and its ability to function properly.
   4. **3 points**: Severe reduction in staining. The staining is severely reduced, showing a significant depletion of proteoglycans in the matrix. The cartilage's mechanical properties are likely to be severely affected, making it more vulnerable to damage.
   5. **4 points**: No staining. There is almost no staining of the cartilage matrix, indicating a near-complete loss of proteoglycans. At this stage, the cartilage has lost much of its normal structure and function.
4. **Tidemark Integrity Scoring**
   1. **0 point**: Intact tidemark. The tidemark, which is a boundary between the uncalcified and calcified cartilage layers, is continuous and undamaged. It plays an important role in maintaining the integrity of the cartilage layers and their interaction with the underlying bone.
   2. **1 point**: Disrupted by vessels. The tidemark has been invaded or disrupted by blood vessels. This vascular invasion can bring inflammatory cells and other factors that can contribute to cartilage degradation and the progression of osteoarthritis.

The total Mankin’s score is the sum of the scores from these four categories. A higher Mankin’s score indicates a more severe degree of cartilage degeneration in osteoarthritis.
