# Supplementary figures and images for: Comparison of passive and active motion: effect of myokine irisin on cartilage in knee osteoarthritis rats
Source: Front Physiol. 2025 Jul 29;16:1639174. doi: 10.3389/fphys.2025.1639174 (PMC12339569; doi:10.3389/fphys.2025.1639174)

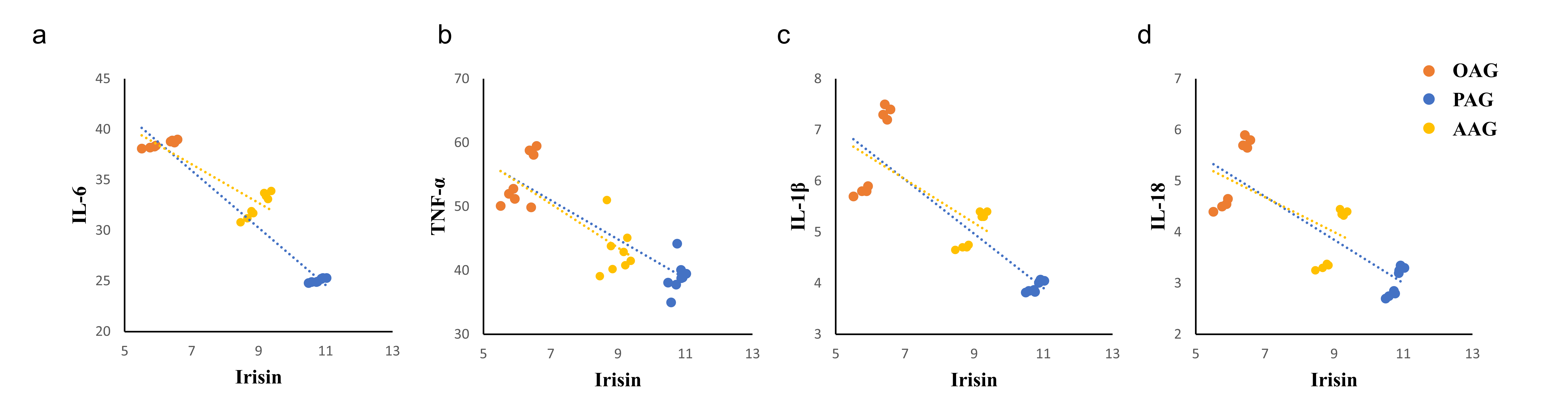

Supplement: Supplementary file 2 [file Image1.tif]
